# Corpus Callosotomy: Editorial

**DOI:** 10.3390/brainsci12081006

**Published:** 2022-07-29

**Authors:** Ayataka Fujimoto, Tohru Okanishi

**Affiliations:** 1 Comprehensive Epilepsy Center, Seirei Hamamatsu General Hospital, Hamamatsu 430-8558, Japan; 2 Division of Child Neurology, Department of Brain and Neurosciences, Faculty of Medicine, Tottori University, Yonago 683-8503, Japan; t.okanishi@tottori-u.ac.jp

Since corpus callosotomy (CC) was first reported in 1940 [1,2], this surgical technique has become one of the most recognized epilepsy surgeries [3]. While this method was temporarily viewed in a negative light, meaning few studies on CC were performed between the 1970s and 2000s, the number of CCs performed and the number of papers published on CC have increased in recent years [4].

One reason why CC is not often performed is because CC is a palliative rather than a radical therapy [5]. This is probably because when considering open cranial surgery for intractable epilepsy, the mainstream process is generally to identify the epileptic focus and perform epileptic focus resection to radically eliminate the source of epileptic seizure. In addition, permanent higher brain function disorders might arise after CC, such as split-brain syndrome [6]. We have performed many CCs and have never experienced typical split-brain phenomena or memory impairment [7]. In addition, temporary postoperative disturbance of consciousness [4] and postoperative chemical meningitis [8] are factors that may cause anxiety for all medical personnel and caregivers involved in the postoperative management of patients who have undergone CC. These reasons might lead to hesitation in proceeding with CC.

However, if the epileptic focus is not identified from stereotactic electroencephalography (SEEG) or intracranial electrode placement following focal resection, is it sufficient to simply remove the electrode? Even though vagus nerve stimulation (VNS) is available as a palliative treatment, VNS therapy for drop attacks can be enhanced by adding VNS therapy to CC [9], but whether this is sufficient to control seizures is unclear. In fact, inadequate efficacy of VNS has been reported [10].

Many studies have compared CC and VNS therapy, but what these methods have in common is simply that they are palliative therapies. In terms of drop attacks, clinicians need to consider whether the drop attack represents epileptic spasm (ES), atonic seizure, or bilateral tonic seizure. However, the efficacy of CC and VNS therapy is often reported without clearly distinguishing among seizure types. Since the mechanisms underlying CC and VNS therapies are completely different, direct comparison between these methods is not appropriate.

The effect of CC on the “drop attack” is marked [2]. ES is found mainly in West syndrome, and adrenocorticotropic hormone (ACTH) therapy is the core treatment. However, if the response to ACTH therapy or anti-seizure medications is insufficient, surgical treatment, such as CC or radical surgery, is considered. The rate of seizure resolution by CC with respect to ES varies, but is reported to be 25–79%. Conversely, the rate of seizure resolution with radical surgery, including cortical resection, lobectomy, and hemispherical disconnection, is 61–83% [11,12,13,14,15,16,17,18,19,20]. The major difference in patient backgrounds between the two surgical options was that 83–100% (mean, 94%) of patients with radical surgery had brain lesions, as shown by magnetic resonance imaging (MRI). Radical surgery is usually performed on a single hemisphere of the brain, and has been considered to have been performed on patients that show signs of lateralization preoperatively. The fact that radical surgery has been performed based on not only MRI, but also nuclear medicine examinations (including positron emission tomography), has been reported [21], along with 70 cases that underwent radical surgery based only on MRI without invasive EEG monitoring [22].

In terms of CC, 0 to 65% (mean, 17%) of cases have brain lesions, less than with radical respective surgery. As a preferable prognostic factor for CC, “no brain lesions” have been reported [20]. Therefore, in the selection of CC for intractable ES, on the one hand, the absence of structural factors is considered a positive factor. On the other hand, successful cases have also been described with bilateral widespread polymicrogyria, cortical/subcortical tubers in tuberous sclerosis complex, and diffuse atrophy [11,13,15,20]. Since selecting a radical operation is difficult for such lesions, CC may be indicated.

In a group of patients with West syndrome without brain lesions, who showed predominantly ES, but also had some tonic seizures, ES was more effectively controlled by CC than tonic seizures [10]. CC is less effective in Lennox-Gastaut syndrome than in West syndrome [23]; therefore, CC may be necessary during the West syndrome stage or the ES-only stage. After CC, if epileptogenicity is lateralized to one hemisphere and clinically focal (asymmetric) ES is exhibited, radical surgery might be considered, and the seizure resolution rate in such cases is 43–71% [11,14,15,18,24].

Based on advanced analyses for presurgical EEG, less power and less connectivity of high-gamma activities on presurgical scalp EEG recordings [14] and lower phase lags in ictal slow and gamma waves among bilateral hemispheres [17] have been associated with favorable seizure outcomes after CC.

Okanishi and Fujimoto have reported that ES can be categorized as follows: (1) focal-onset ES; (2) potentially focal-onset ES; (3) generalized onset/bilateral focal-onset ES with low callosal modulation; and (4) generalized onset. The generalized onset occurs in the pathology of ES with high callosal modulation. Among these, CC is highly effective against generalized onset ES. Pathologies in which the corpus callosum is strongly involved in the development of ES and bilateral synchronization are often observed in generalized onset ES. In a pathology where a seizure starts from one hemisphere, but clinically manifests as generalized ES via the corpus callosum, CC may lead to lateralization and focal ES. In such cases, subsequent radical surgery might be indicated [2].
